# Barriers in access to healthcare services for individuals with disorders of sex differentiation in Bangladesh: an analysis of regional representative cross-sectional data

**DOI:** 10.1186/s12889-020-09284-2

**Published:** 2020-08-18

**Authors:** Alam Khan, T. M. Fahad, Md Imran Nur Manik, Hazrat Ali, Md. Ashiquazzaman, Md Ibrahim Mollah, Tanjeena Zaman, Md Shariful Islam, Moizur Rahman, Aminur Rahman, Mostafizur Rahman, Tarannum Naz, Mahmud Arif Pavel, Md. Nuruzzaman Khan

**Affiliations:** 1 Department of Pharmacy, University of Rajshahi, Rajshahi, 6205 Bangladesh; 2 Department of Molecular Medicine, The Scripps Research Institute, Florida, USA; 3 Department of Pharmacy, Northern University Bangladesh, Dhaka, Bangladesh; 4 Department of Pharmacy, International Islamic University Chittagong, Chittagong, Bangladesh; 5 Department of Fisheries, University of Rajshahi, Rajshahi, Bangladesh; 6 Department of Biology, University of Hail, Hail, Kingdom of Saudi Arabia; 7 Department of Veterinary and Animal Sciences, University of Rajshahi, Rajshahi, Bangladesh; 8 Department of Population Science and Human Resource Development, University of Rajshahi, Rajshahi, Bangladesh; 9 Department of Genetic Engineering and Biotechnology, University of Dhaka, Dhaka, Bangladesh; 10 Department of Population Sciences, Jatiya Kabi Kazi Nazrul Islam University, Mymensingh, Bangladesh

**Keywords:** Disorder of sex development, Social discrimination, Healthcare facilities, Bangladesh

## Abstract

**Background:**

Worldwide people in disorder of sex development (DSD) faces multiple barriers while seeking their social rights, particularly healthcare services. We aimed to explore the healthcare opportunities available to them, using patterns of healthcare utilization and difficulties faced by DSD population in accessing healthcare services in Bangladesh.

**Methods:**

Data from a total of 945 DSD population and 71 medical staff were analyzed, collected from three major divisions (Dhaka, Chittagong, and Rajshahi) in Bangladesh during the period of January to December of 2017. A structured questionnaire was used to collect data via face-to-face interviews. Descriptive statistic was used to determine the frequencies of the visit by the DSD population in healthcare facilities as well as to analyze difficulties experienced by the DSD population in getting healthcare services. Multivariate regression analysis was used to explore the association between perceived barriers in getting healthcare services and failures of the DSD population to receive the healthcare services.

**Results:**

Present data revealed that around 80% of DSD population sought healthcare services from government healthcare facilities, where the overall success rate in getting healthcare services was less than 50%. The DSD population reported a number of reasons for failures in getting healthcare services, including non-friendly interaction by non-clinical hospital’s staff, non-friendly interaction by physicians, public fright as general people do not want to mingle with a DSD person, undesirable excess public interest in DSD individuals, and limitation of the treatment opportunities of hospitals to merely male or female patients. Among the stated reasons, the most frequently reported reason was non-friendly interaction by physicians (50.27%), followed by undesirable excess public interest in DSD individuals (50.16%).

**Conclusion:**

DSD population in Bangladesh have limited access to healthcare facilities and facing multiple barriers to get healthcare services. Initiatives from the government and social organizations are important to ensure their access to healthcare services.

## Background

Sex development disorders are considered as a heterogeneous group of abnormalities characterized by an incongruence of gonadal, chromosomal, and genital development [1, 2]. It is conventionally named as ‘intersex’, ‘transgender’, ‘hermaphrodite’, and ‘pseudohermaphrodite’. However, a broader term “disorders of sex differentiation (DSD)” widely used to refer all kind of sexual disorders, was proposed in the Chicago Consensus held in 2005 [1, 3–6]. The prevalence of true DSD is low and approximately only one in every 4500–5500 population [7, 8]. However, this rate might increase to one in 300 population if all forms of congenital anomalies are considered, such as one or both undescended testes in the scrotum (cryptorchidism) and anomalies in the opening of the urethra on the penis (hypospadias) [1, 9–11]. The actual occurrence of DSD is much higher in developing countries than in developed countries. For instance, the prevalence of ambiguous genitalia in Saudi Arabia and Egypt were found to be one in every 2500 and 3000 live births, respectively [12, 13]. On the other hand, a study in Germany identified four times higher occurrence of the congenital disorder among newly born infants of non-German parents as compared to infants of German parents [14]. This is considered to be associated with greater rates of consanguinity in the migratory inhabitants, as they have the autosomal recessive inheritance of numerous conditions of DSD [14].

The DSD population in developing countries, including Bangladesh are often deprived of social facilities, including marriage, employment, and even often rejected by their parents [15–17]. Being usually called “Hijra”, the government of Bangladesh officially recognized them as the third gender since 2011 [18]. Since then the Ministry of Social Welfare in Bangladesh recorded around 10,000 DSD population. However, both the government registered DSD’s organization (Badhan Hijra Sangha) and non-governmental organizations (NGOs) have refused this number. According to their estimates, the actual number of the DSD population in Bangladesh should be between 30,000 and 150,000, of which around 10,000 DSD population live only in Dhaka division [19, 20]. The vulnerability of the DSD population in Bangladesh in terms of human basic needs including health has been reported by both the government and non-government organizations, calling for special programs to improve their lifestyle and social security. However, to date research on the DSD population is very limited in Bangladesh. There are a few studies that focused mainly on social issues including education, marital status, living status, lifestyles and religion [17, 18, 20]. They are also more vulnerable to health issues, such as infection, tumor development, mental health and premature death [10, 17, 21]. Prevention of these adverse outcomes requires special healthcare services. However, often this issue is overlooked in Bangladesh. We are not aware of any study that addresses this healthcare issue. Therefore, in this study, an attempt has been made to explore healthcare utilization by the DSD population in Bangladesh and the problems they faced in receiving healthcare services. Medical doctors were also asked about problems they faced in providing services to the DSD population (mostly institutional level problems). Findings will be helpful for the policymakers in making evidence-based policies to overcome different sorts of difficulties that the DSD population faces in getting healthcare services in Bangladesh.

## Methods

### Study area

Data for this study were collected from Dhaka, Chittagong, and Rajshahi divisions, which are the major divisions of Bangladesh located in the middle, east, and west side of the country, respectively. Because of increasing economic opportunities, administrative activities and educational purposes, these three divisions are places of livelihood for millions of people. Most of the DSD population in Bangladesh have escaped from their parental family and live with other DSD population as groups (known as DSD/transgender/third gender community or Hijra sangha in local language) in places where a large number of the population gathers for economic activities. The majority of these groups, such as (a) Laila Hijra sangha in Shampur, Dhaka, (b) Bukul Hijra sangha in Dhamri, Dhaka, (c) Hamida Hijra sangha in Shamoli, Dhaka (d) Kali Hijra sangha in Savar, Dhaka were non-registered communities operated through a group leader from among themselves who is called “Guru” sometimes “Guruma”. In addition, a government registered DSD community, named ‘Badhan Hijra Sangha (BHS)” is also located in this division. Similar non-registered DSD communities are also located in Chittagong and Rajshahi divisions such as Jorna Hijra sangha in Bandar, Chittagong, Juli Hijra sangha in Railgate, Rajshahi, likewise operated through a group leader from among themselves.

### Study design

A cross-sectional survey was conducted among DSD individuals who live within DSD communities as well as with parental families and life partners in the selected regions, from January to December of 2017. DSD individuals in Bangladesh can be considered as a hidden population, living in different regions, outside the parental family with other DSD individuals as a group. They seldom live in their parental family or with their life partner. They were primarily identified using the birth registration book of the ward office in each study area. The government ward office of Bangladesh usually functions to conduct birth/death registration as well as to provide nationality certificate. Individuals whose sexual status was ‘third gender’ or ‘Hijra’ in birth registration books were identified. The locations of DSD communities or Hijra sangha in each study area were determined using the living addresses (updated) of identified individuals. Although, initial living addresses of DSD individuals are their parental homes, the addresses are often updated subsequently when they left the parental homes and ward office reissue their nationality certificates.

Delivery of a DSD newborn is a matter of shame for the parental family as DSD individuals often faced social discrimination. Hence, some parents of DSD individuals do not register their children in the ward office. Moreover, some DSD individuals morphologically close to either male or female (particularly at childhood when the genital disorder was not identified) are registered accordingly. Such DSD individuals often live with their parental family and were identified using information received from primarily identified DSD individuals. The survey was also conducted among hospital/clinic’s staff in hospitals/clinics where DSD individuals usually seek treatment.

### Sampling design

The survey was conducted in two stages. At the first stage, ten DSD communities in the Dhaka division, three DSD communities in the Chittagong division and four DSD communities in the Rajshahi division were selected using simple random sampling. At the second stage, through another simple random sampling, the total 945 DSD population were interviewed. Among the DSD population, 447, 228 and 270 DSD individuals were interviewed from Dhaka, Chittagong and Rajshahi divisions, respectively. Moreover, public healthcare facilities were selected based on the DSD population responses, whereas, 71 medical doctors were interviewed from 24 public healthcare facilities. Among public hospitals, 10, 7 and 7 hospitals were selected through simple random sampling from Dhaka, Chittagong and Rajshahi divisions, respectively. The share of medical doctors of Dhaka, Chittagong, and Rajshahi division were 32 (45.1%), 18 (25.35%), and 21 (29.58%), respectively.

### Sample size

The required number of population sample was determined using formula $n=p\kern0.32em \left(1-p\right)\times {\left(\frac{Z}{E}\right)}^2$ [22], where, p is the sample proportion (0.5), E is the margin of error (0.04), Z is the critical value (1.96), and α is the significance level (0.05). The study sample comprised of 544, 325, and 338 DSD populations for Dhaka, Chittagong, and Rajshahi division, respectively. The non-response rates were 17.83% (97 out of 544) for Dhaka, 29.85% (97 out of 325) for Chittagong, and 20.12% (68 out of 338) for Rajshahi division. The questionnaire that was partially filled was not considered, instead included within non-response data. Non-response data were excluded from the analysis.

### Data collection

The members of the selected DSD communities as well as DSD individuals living with parental families and life partners were interviewed through face-to-face interview using structured questionnaires. The questionnaires used in this study were developed in two-stages. An initial version of the questionnaire was developed by reviewing related literature and following the World Bank guideline for designing and conducting a household survey [23]. This initial version of the questionnaire was in English language and was translated in Bengali language for easier understanding by the DSD population. A validation survey was then conducted by interviewing 15 DSD individuals from three selected divisions (5 from each division). Differences reported were adjusted in the pre-designed questionnaire. This adjusted version of the questionnaire was then submitted for ethical approval from the Rajshahi University Ethical Council (RUEC) of Bangladesh (approval number RUEC/2015/Hijra001). The suggestions of the members of the Ethics committee on the submitted version of the questionnaire were then incorporated to develop the final version of the questionnaire. Questions used include: ‘how many times you have tried to get treatment in government hospitals from January to December of 2015?’, ‘how many times you succeed in getting treatment in government hospitals during the same date?’, ‘how many times you have tried to get treatment in private hospitals from January to December of 2015?’, ‘how many times you succeed in getting treatment in private hospitals during the same time?’ (Questionnaire attached as supplementary file 1). Moreover, in the questionnaire, DSD individuals were requested to select some perceived reasons of not getting care in hospitals/clinics (as barriers or difficulties in successful receiving healthcare services), that they experienced during their trial for healthcare services, such as ‘non-friendly interaction by hospital’s staff’, ‘non-friendly interaction by physicians’, ‘public fright as general people do not want to stand in line before or after DSD person’. In addition to these perceived barriers, in the questionnaire, DSD individuals had an option to mention extra difficulties in getting healthcare services (if they experienced) (supplementary file 1). No additional barrier was reported by them. A separate questionnaire was used for collecting data from healthcare providers which was taken through similar stages that we used for developing the DSD questionnaire (Questionnaire attached as supplementary file 2). It is better to mention, both the questionnaires were developed for the purposes of this study and have not been published elsewhere.

### Study variables

Several forms of difficulties experienced by the DSD population were considered as study variables. These were; (i) non-friendly interaction by non-clinical hospital’s staff (Barrier 1); (ii) non-friendly interaction by physicians (Barrier 2); (iii) public fright as general people do not want to stand in line before or after DSD person (Barrier 3), (iv) undesirable excess public interest as general people congregate around DSD person (because of exceptional appearance, gesture and voice of DSD individuals) (Barrier 4) and (v) limitation of the treatment opportunities of hospitals to merely male or female patients (Barrier 5). These difficulties were studied along with DSD population’s socio-demographic characteristics, including age, and educational status. Healthcare services provider’s (doctors) perceptions in providing healthcare services to the DSD population were also collected.

### Statistical analysis

Descriptive statistic was used to characterize the demographic profile of the DSD population as well as to analyze difficulties experienced by the DSD population in getting healthcare services and difficulties faced by medical staff in providing services to DSD individuals. Multivariate regression analysis was used to identify significant barriers where the number of failures in getting access to healthcare services was considered as the dependent variable. The Statistical Package for Social Sciences (SPSS) version 20 software (IBM SPSS Inc., Chicago, IL, USA) was used for statistical analysis.

## Results

### Living arrangement of DSD population

**Fig. 1:**
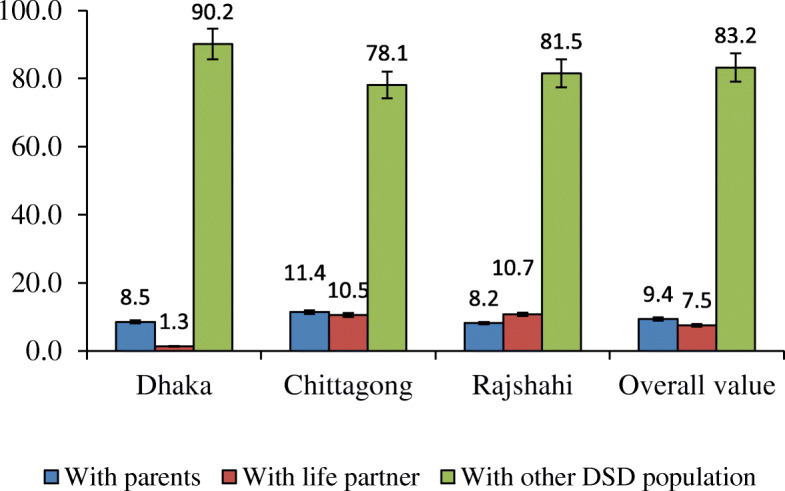
Distribution of DSD population in Bangladesh by people with whom they normally live (in percentage)

In Bangladesh, people in mainstream societies usually live together as a joint family with husband-wife, children, parents, and grandparents (if alive). In comparison, it was found that most of the DSD population in Dhaka (90.2%), Chittagong (78.1%), and Rajshahi (81.2%) divisions live outside of their original parental home. They live together with other DSD population in groups named as Hijra sangha (Bengali language)/DSD community (Fig. 1). Only a minor portion of them (9.3%) found were live with parents, and another trivial portion of the DSD population (7.5%) were live with their life partners. In Chittagong and Rajshahi divisions, proportions of the DSD population living with life partners were nearly similar, around 10% of the DSD individuals were live with their life partners. On the other hand, in the Dhaka division, merely 1.3% of the DSD population were live with life partners (Fig. 1). The mean age of the DSD population participated in current data collection were 35, 32 and 27 years for Dhaka, Chittagong and Rajshahi divisions, respectively (Table 1). Major DSD population in Bangladesh were illiterate (64.4–70%) (Table 1).

**Table 1:** Age and educational characteristics of the DSD population in Bangladesh

Characteristics	Dhaka	Chittagong	Rajshahi
Age			
Age range (Min-Max)	20–61	18–47	17–43
Mean Age (years)	35	32	27
Education			
Illiteracy (%)	70.0	70.6	64.4
Primary education (%)	16.3	25.4	24.8
Secondary education (%)	11.6	2.6	8.9
Higher education (%)	2.0	1.3	1.9

Min: The minimum age in years, Max: The maximum age in years

### Access of DSD population in government and private healthcare facilities

The frequencies of attempts made by the DSD population to get treatment in government and private healthcare facilities for the years 2015 and 2016 were compared. The results are shown in Table 2. The majority of the DSD population were reported that they in most cases have sought treatment from government healthcare facilities (81.62% for the year 2015, and 80.42% for the year 2016). Division-wise outcomes showed that such percentages were comparatively high in the Dhaka division (85.56 and 82.45% for 2015 and 2016, respectively), followed by Rajshahi (80.92 and 78.57% for 2015 and 2016, respectively), and Chittagong (78.38 and 80.23% for 2015 and 2016, respectively) divisions.

**Table 2:** Frequencies of the visit by DSD population in government and private health care facilities in three major divisions of Bangladesh

Years	Divisions	Total visits in Govt. HCF	Total visits in Pvt. HCF	% visits in Govt. HCF	% visits in Pvt. HCF
2015	Dhaka	1161	196	85.56	14.44
	Chittagong	580	160	78.38	21.62
	Rajshahi	827	195	80.92	19.08
	Overall percentage			82.33	17.66
2016	Dhaka	1198	255	82.45	17.55
	Chittagong	564	139	80.23	19.77
	Rajshahi	704	192	78.57	21.43
	Overall percentage			80.79	19.20

Note: The total number of respondents were 447, 228, and 270 in Dhaka, Chittagong and Rajshahi divisions, respectively. The non-response rates were 17.83% (97 out of 544), 29.85% (97 out of 325), and 20.12% (68 out of 338) in Dhaka, Chittagong and Rajshahi divisions, respectively. Govt. HCF: Government health care facilities, Pvt. HCF: Private health care facilities,

**Fig. 2:**
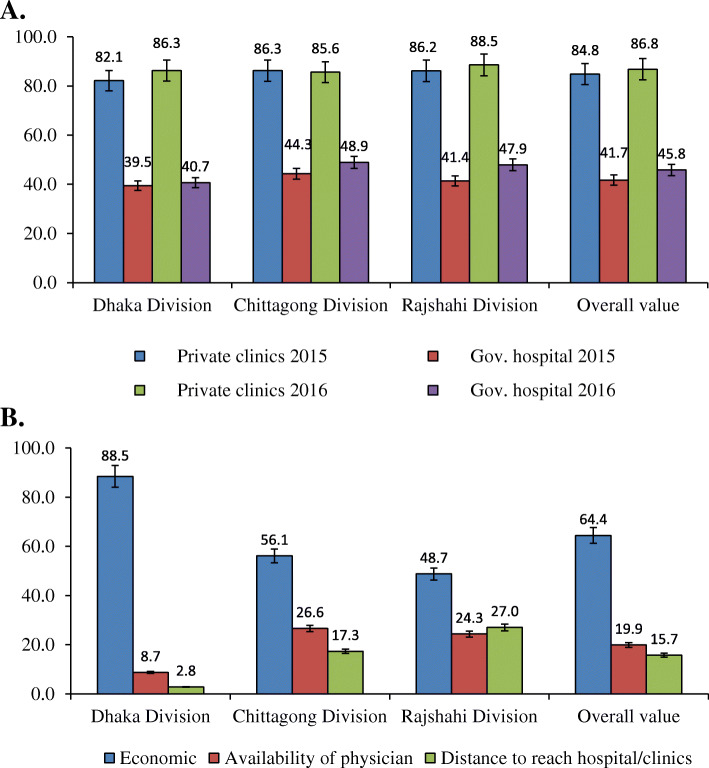
Access of DSD population in healthcare services in Dhaka, Chittagong and Rajshahi divisions of Bangladesh. **a** Success rates (in percentage) of the DSD population in receiving healthcare services in government and private healthcare facilities. **b** Reasons that motivate the DSD population to prefer government healthcare facilities (in percentage)

It was found that government healthcare service providers frequently refused to provide healthcare services to DSD population. However, a significant and reasonable rate of successfully receiving healthcare services from private healthcare facilities (more than 80%) were noticed for both 2015 and 2016 (Fig. 2a). In contrast, the accessibility of the DSD people to healthcare services in government healthcare facilities was always found to be below 50%. The success rates in getting healthcare services from government hospitals in Chittagong and Rajshahi divisions were higher in 2016 than in 2015, however, the rates were still below 50% (Fig. 2a). DSD individuals experienced a number of barriers in getting healthcare services from both government and non-government hospitals and clinics. Most cited barriers were non-friendly interaction by physicians (Barrier 2; 50.27%) followed by the undesirable excess public interest in DSD individuals (Barrier 4; 50.16%), non-friendly interaction by non-clinical hospital’s staff (Barrier 1; 46.67%), limitation of the treatment opportunities of hospitals to merely male or female patients (Barrier 5; 26.88%), and public fright as general people do not want to mingle with a DSD person (Barrier 3; 26.14%) (Table 3). The multivariate analysis showed that non-friendly interaction by non-clinical hospital’s staff (Barrier 1, *p =* 0.005), non-friendly interaction by physicians (Barrier 2, *p* = 0.006), public fright as general people do not want to mingle with DSD person (Barrier 3, *p* = 0.000), and undesirable excess public interest toward DSD individuals (Barrier 4, *p* = 0.013) were statistically significantly associated with a decrease in the number of failures to get care from the government healthcare facilities (Table 4). Further regression analysis considering individual division found that some barriers were significant in a particular division only (Table 5). Non-friendly interaction by physicians (Barrier 2) in Dhaka division, non-friendly interaction by non-clinical hospital’s staff (Barrier 1) and limitation of the treatment opportunities of hospitals to merely male or female patients (Barrier 5) in Chittagong division, and undesirable excess public interest as general people congregate around DSD persons (Barrier 4) and limitation of the treatment opportunities of hospitals to merely male or female patients (Barrier 5) in Rajshahi division were statistically significantly associated with the decrease in the number of failures to get care. On the other hand, public fright as general people do not want to mingle with DSD person (Barrier 3) in Chittagong division was statistically significantly associated with an increase in the number of failures to get care in healthcare facilities (Table 5). The minimum number and the maximum number of trials that were experienced by the DSD population to get care in healthcare centers, including the minimum and the maximum number of failures are presented in Table 6.

**Table 3:** Barriers reported by the DSD population in getting access to health care services, in three major divisions of Bangladesh

#	Discouraging Interactions	Dhaka (%)	Chittagong (%)	Rajshahi (%)	Overall (%)
1	Non-friendly interactions by non-clinical hospital’s staff (Barrier 1)	30.87	63.16	58.89	46.67
2	Non-friendly interaction by physicians (Barrier 2)	34.28	68.42	61.48	50.27
3	Public fright as general people do not want to mingle with DSD person (Barrier 3)	22.37	47.37	14.44	26.14
4	Undesirable excess public interest as general people congregate around DSD person (Barrier 4)	43.40	58.77	54.07	50.16
5	Limitation of the treatment opportunities of hospitals to merely male or female patient (Barrier 5)	20.58	19.74	43.33	26.88

Note: Total percentages are over 100%, due to the multiple response variables. The total number of respondents were 447, 228, and 270 in Dhaka, Chittagong and Rajshahi divisions, respectively. The non-response rates were 17.83% (97 out of 544), 29.85% (97 out of 325), and 20.12% (68 out of 338) in Dhaka, Chittagong and Rajshahi divisions, respectively

**Table 4:** Overall outcomes of multivariate regression analysis where the number of failures in receiving healthcare services was considered as dependent variable

Independent variables	Unadjusted Coefficient	Adjusted Coefficient	t-values	*p*-values	Percentage of failures (Number of failures/Number of trials)
Barrier 1	−0.065	− 0.271	−2.842	0.005	57.81 (629/1088)
Barrier 2	−0.063	− 0.205	−2.743	0.006	55.78 (1249/2239)
Barrier 3	−0.080	− 0.288	−3.511	0.000	56.91 (1194/2098)
Barrier 4	−0.057	−0.195	−2.487	0.013	56.21 (1390/2473)
Barrier 5	−0.038	−0.164	−1.666	0.096	55.86 (791/1416)

Note: Numbers within parentheses of the left terminal column indicating the total number of failures/total number of trial. t-value is the t-statistics. Unadjusted coefficient: Not adjusted with any covariates. Barrier 1: non-friendly interactions by non-clinical hospital’s staff; Barrier 2: non-friendly interaction by physicians; Barrier 3: public fright as general people do not want to mingle with DSD person; Barrier 4: undesirable excess public interest as general people congregate around DSD person; Barrier 5: limitation of the treatment opportunities of hospitals to merely male or female patient

**Table 5:** Division-wise outcomes of multivariate regression analysis where the number of failures in receiving healthcare services was considered as dependent variable

Division	Independent variables	Unadjusted Coefficient	Adjusted Coefficient	t-values	*p*-values	Percentage of failures (Number of failures/Number of trials)
Dhaka	Barrier 1	−0.045	−0.144	−1.160	0.246	61.67 (280/454)
	Barrier 2	−0.102	−0.279	−2.662	0.008	59.23 (398/672)
	Barrier 3	0.067	0.191	1.749	0.081	59.08 (361/611)
	Barrier 4	0.070	0.176	1.811	0.071	59.56 (536/900)
	Barrier 5	0.065	0.168	1.703	0.089	54.53 (259/475)
Chittagong	Barrier 1	−0.037	−0.149	−0.921	0.031	55.14 (252/457)
	Barrier 2	−0.066	−0.195	−1.634	0.103	54.53 (337/618)
	Barrier 3	0.092	0.336	2.283	0.023	54.38 (323/594)
	Barrier 4	0.055	0.195	1.346	0.179	54.07 (379/701)
	Barrier 5	−0.096	−0.358	−2.364	0.018	58.77 (134/228)
Rajshahi	Barrier 1	0.030	0.095	0.743	0.458	54.80 (97/177)
	Barrier 2	−0.037	−0.114	−0.927	0.354	54.16 (514/949)
	Barrier 3	0.012	0.037	0.293	0.069	57.11 (510/893)
	Barrier 4	−0.131	−0.739	−3.314	0.001	54.47 (475/872)
	Barrier 5	−0.078	−0.206	−1.959	0.041	55.82 (398/713)

Note: Numbers within parentheses of the left terminal column indicating the total number of failures/total number of trial. t-value is the t-statistics. Unadjusted coefficient: Not adjusted with any covariates. Barrier 1: non-friendly interactions by non-clinical hospital’s staff; Barrier 2: non-friendly interaction by physicians; Barrier 3: public fright as general people do not want to mingle with DSD person; Barrier 4: undesirable excess public interest as general people congregate around DSD person; Barrier 5: limitation of the treatment opportunities of hospitals to merely male or female patient

**Table 6:** The minimum number and the maximum number of both trials and failures to get healthcare services in three major divisions of Bangladesh

Divisions	Number of trials			Number of failures	
	Minimum	Maximum		Minimum	Maximum
Dhaka	1	9		1	6
Chittagong	1	11		0	7
Rajshahi	2	9		0	6
Overall	1	11		0	7

On the other hand, hospital staff have reported a number of difficulties that they faced in treating DSD population. For instance, paper documents which are supplied by the hospital’s authority merely consider either male or female patient and there is no option for the third gender (Table 7).

**Table 7:** Difficulties faced by the healthcare providers in government hospitals in treating DSD population in three major divisions of Bangladesh

#	Difficulties	Dhaka (%)	Chittagong (%)	Rajshahi (%)	Overall (%)
1	All papers documents supplied by the hospital’s authority merely consider either male or female patient.	33.23	42.64	28.71	34.85
2	Some physicians do not prefer to treat DSD patients	11.34	10.61	12.57	11.51
3	General people gather around DSD patient which hinder hospital management.	10.14	15.48	23.93	16.51
4	DSD patients enter hospitals with an excess number of companions (DSD persons) which hinder hospital management.	12.61	10.33	9.62	10.84
5	Hospital’s staff suspect that DSD population enter the hospital to ask for charity instead of illness issues.	32.73	21.01	25.20	26.30

## Discussion

The study was conducted to explore the treatment opportunity of the DSD population in government and private healthcare facilities and to identify the problems that they faced in getting the services. DSD population have limited access to healthcare services. Non-friendly interaction by physicians and non-clinical hospital’s staff, public fright as general people do not want to mingle with DSD individuals as well as undesirable excess public interest toward DSD individuals were reported as the major problems in getting healthcare services in Bangladesh. These findings are unique and could help to develop policies to ensure healthcare services for the DSD population in Bangladesh.

We have found a high rate of illiteracy among the DSD individuals. Criticism by the general people, misbehaviour and misunderstanding by classmates, teachers, and people of mainstream society are the causes of such lower rate of literacy [17, 18]. Most of the DSD population in Bangladesh live in slums, together with other DSD population. However, a trivial portion of them live in the original parental home with parents or in a rental home with a life partner. Previous studies conducted in Bangladesh found such exclusion from the parental family was associated with the criticisms of their neighbours, discrimination by their parental family, exclusion from social rights and attraction by the members of Hijra Sangha [17, 18]. However, a higher number of the DSD population in developed countries live with parents, life partners or alone. For instance, 30.3 and 28.6% of the total DSD population in Germany were live alone and with their parents, respectively [2]. The rates were even lower in France, Poland, and the Netherlands whereas around 48, 62, and 46% of the total DSD population, respectively, were married or live with a life partner [2]. Such a higher rate of exclusion of the DSD population from mainstream societies in Bangladesh are associated with depression, frustrations, and insecurities, which make them more vulnerable to illness that demands enough healthcare services [17, 18, 24–26].

The present investigation found that the majority of the DSD population uses governmental healthcare facilities for getting healthcare services. Treatment in the government healthcare facilities is associated with low cost which was the principal reason for which they often have tried to get treatment in governmental hospitals. Other reported causes of choosing governmental healthcare facilities were the feasible distance and availability of physicians (Fig. 2b). Unfortunately, the entrance of the DSD population to government healthcare services were often restricted and DSD patients experienced a number of difficulties. In regression analysis, some difficulties were ended up with negative coefficients. The findings of a negative association or lack of an association between the number of failures and some barriers might be due to a lower number of trials to access healthcare services because the question on reasons for failure was asked only to those who have tried at least once and failed at least once. In our overall data, success rates to get healthcare services by the DSD population in government healthcare facilities always found below 50%, and the rate was above 80% in private healthcare facilities. This suggests their entrance in healthcare facilities are restricted, particularly in government healthcare facilities. However, such entrance limitation might due to the higher number of patients from mainstream societies as well as restrictions by hospital authorities, and/or physicians. As found in other studies, as well as by this study, patients in mainstream societies did not feel comfortable in receiving healthcare services at the same time of DSD population, which might lead hospital authorities, and/or physicians to impose restriction in getting healthcare services for DSD population [16–18]. Administrative policies of healthcare facilities also impose barriers. For example, administrative documents were designed for either male or female patients only, where DSD patient have no option to report. Moreover, in some cases, a physician from the government and private healthcare organizations do not prefer to treat DSD population because of limited understanding of culture, gender, and sexuality of DSD patients [17]. Other mentioned difficulties include, people of mainstream society gather around DSD patient which hinder hospital management, DSD patient enter into hospital with a large number of accompanies (DSD persons) which also hinder hospital management, and some staff of hospitals suspect that DSD individuals were entering to the healthcare facilities to ask for charity instead of illness issues.

These difficulties limit them from getting healthcare services, which is a violation of basic human rights and reducing the quality of life, as well as might be a cause of other adverse consequences. For instance, restrictions in getting healthcare services from governmental/non-governmental sources often lead them to self-treatment by collecting medicine from a pharmacy shop without consulting any physician. It is worth mentioning that any person can buy most of the drugs (even antibiotics, steroids, CNS depressants, and stimulants) from pharmacies without a prescription in Bangladesh [26–28]. Such self-treatment might contribute to receiving the wrong medications, leading to the occurrences of adverse outcomes including drug resistance [26, 27]. Country-level policies to ensure their access to healthcare services are therefore important. Adding a third gender option or DSD population option in the hospital’s paper documents, and proper direction to the hospital authorities and physicians to treat the DSD population are imperative to overcome administrative restrictions of the hospital and/or clinics. Mass media campaigns and curriculum-based education are often used to influence the social behaviour of the entire population [29–31]. In Ghana, population and family life learning is an integral component of social studies at the basic school level [31]. The Intersex Campaign for Equality (IC4E) is an organization that works to campaign for equality of human rights of the DSD population in the USA [32]. Campaigns through the newspapers, electronic, and social media about the right of the DSD population are also important for changing the perception of the population in the mainstream societies in Bangladesh. It is also recommended to integrate the concept of diversity (DSD population) in the school textbooks. There is no peer support group (PSG) for DSD patients in Bangladesh. So it is crucial for clinicians to work in collaboration with existing international PSGs {such as www.dsdfamilies.org (UK), www.accordalliance.org (USA), www.dsdgenetics.org (Australia)} to progress the quality of outcomes in the DSD population through improved education, healthcare, and research.

### Strength and limitations

This study has several strengths and some limitations. This is the first study in Bangladesh that explore the difficulties faced by the DSD population in getting healthcare services. Moreover, the data were collected from quite a large number of DSD population and medical doctors covering three major divisions in Bangladesh where the majority of the DSD communities are located.

Although most of the sampling such as DSD communities selection, interviewing DSD individuals in DSD communities, healthcare facilities selection, interviewing staff of healthcare facilities were carried out using simple random sampling, however, some hidden DSD individuals particularly those who live with parental families were identified through snowballing. Moreover, this study did not consider details social factors and economic condition of DSD individual in the analysis that could restrict the DSD population in accessing healthcare services. Additionally, all the data we have analyzed were self-reported by the DSD population or the medical doctors with no scope of validation by the interviewers.

## Conclusions

DSD population in Bangladesh are facing multiple barriers in getting healthcare services from the government, and private healthcare facilities. The barriers were more prominent in government hospitals than that of private healthcare facilities. Government and social organizations should take initiatives to ensure their rights of getting healthcare services, as well as their rights in the family and society. Awareness should be raised among the general people, communities, and societies to ensure their rights.

## Supplementary information

**Additional file 1.** DSD questionnaire (DSD population interviewed). Disorder sex development (DSD) population were interviewed using questions of this questionnaire.

**Additional file 2.** DSD questionnaire (Medical staff interviewed). Healthcare services provider’s (physicians) perceptions in providing healthcare services to the DSD population were collected using questions of this questionnaire.

## Data Availability

The datasets was generated during the data collection and data analysis are not publicly available. To obtain datasets for further analysis please contact the Corresponding Author.

## Abbreviations

DSD: Disorders of sex differentiation
NGO: Non-government organization
PSG: Peer support group
CNS: Central nervous system
UK: United Kingdom
USA: United States of America

## References

[1] Ganie Y, Aldous C, Balakrishna Y, Wiersma R (2017). Disorders of sex development in children in KwaZulu-Natal Durban South Africa: 20-year experience in a tertiary Centre. J Pediatr Endocrinol Metab.

[2] Röhle R, Gehrmann K, Szarras-Czapnik M, Grinten HC, Pienkowski C, Bouvattier C (2017). Participation of adults with disorders/differences of sex development (DSD) in the clinical study DSD-LIFE: design, methodology, recruitment, data quality and study population. BMC Endocr Disord.

[3] Hughes IA, Houk C, Ahmed SF, Lee PA, LWPES consensus group ESPE consensus group (2006). Consensus statement on management of intersex disorders. Arch Dis Child.

[4] Houk CP, Hughes IA, Ahmed SF, Lee PA; Writing Committee for the International Intersex Consensus Conference Participants Summary of consensus statement on intersex disorders and their management International Intersex Consensus Conference Pediatrics 2006;118:753–757. 10.1542/peds.2006-0737 16882833

[5] Lee PA, Houk CP, Ahmed SF, Hughes IA; International Consensus Conference on Intersex organized by the Lawson Wilkins Pediatric Endocrine Society and the European Society for Paediatric Endocrinology. Consensus statement on management of intersex disorders. International Consensus Conference on Intersex. Pediatrics. 2006;118:e488–e500. 10.1542/peds.2006-0738 16882788

[6] Hughes IA (2008). Disorders of sex development: a new definition and classification. Best Pract Res Clin Endocrinol Metab.

[7] Hamerton JL, Canning N, Ray M, Smith S (1975). A cytogenetic survey of 14,069 newborn infants. Incidence of chromosome abnormalities. Clin Genet.

[8] Blackless M, Charuvastra A, Derryck A, Fausto-Sterling A, Lauzanne K, Lee E (2000). How sexually dimorphic are we? Review and synthesis. Am J Hum Biol.

[9] Bashamboo A, McElreavey K (2014). Consanguinity and disorders of sex development. Hum Hered.

[10] Lee PA, Nordenström A, Houk CP, Ahmed SF, Auchus R, Baratz A (2016). Global disorders of sex development update since 2006: perceptions. Approach Care Horm Res Paediatr.

[11] Nordenvall AS, Frisen L, Nordenstrom A (2014). Lichtenstein, Norenskjold A. Population based nationwide study of hypospadias in Sweden, 1973 to 2009: incidence and risk factors. J Urol.

[12] Abdullah MA, Katugampola M, al-Habib S, al- Jurayyan N, al-Samarrai A, Al-Nuaim A, et al. Ambiguous genitalia: medical, socio-cultural and religious factors affecting management in Saudi Arabia. Ann Trop Paediatr 1991;11:343–348. 10.1080/02724936.1991.11747526 1721791

[13] Mazen I, Hiort O, Bassiouny R, El-Gammal M (2008). Differential diagnosis of disorders of sex development in Egypt. Horm Res.

[14] Thyen U, Lanz K, Holterhus PM, Hiort O (2006). Epidemiology and initial management of ambiguous genitalia at birth in Germany. Horm Res.

[15] Bajpai M (2014). Disorders of sex development: the quintessence of perennial controversies. J Indian Assoc Pediatr Surg.

[16] Khan SI, Hussain MI, Gourab G, Parveen S, Bhuiyan MI, Sikder J (2008). Not to stigmatize but to humanize sexual lives of the transgender (Hijra) in Bangladesh: condom chat in the AIDS era. J LGBT Health Res.

[17] Khan SI, Hussain MI, Parveen S, Bhuiyan MI, Gourab G, Sarker GF (2009). Living on the extreme margin: social exclusion of the transgender population (Hijra) in Bangladesh. J Health Popul Nutr.

[18] Safa N (2016). Inclusion of excluded: integrating need based concerns of hijra population in mainstream development. Soc Anthropol.

[19] Badhan Hijra Sangha, An organization for DSD population in Bangladesh, registered under Ministry of Social welfare of Bangladesh, 2014.

[20] Habib T. A long journey towards social inclusion: initiatives of social workers for hijra population in Bangladesh. Master’s thesis, University of Gothenburg, Sweden. 2012. p. 15. https://gupea.ub.gu.se/bitstream/2077/32545/1/gupea_2077_32545_1.pdf. (Last Accessed 26 Jan 2019).

[21] Grossman AH, D’Augelli AR (2006). Transgender youth: invisible and vulnerable. J Homosex.

[22] Cochran WG (1977). Sampling techniques.

[23] Grosh M, Glewwe P. Designing household survey questionnaires for developing countries. The World Bank documents, Vol 1, Oxford publisher, 2000. http://documents.worldbank.org/curated/en/452741468778781879/pdf/multi-page.pdf. (Last Accessed 26 Jan 2019).

[24] Abela JRZ, Hankin BL, Haigh EAP, Adams P, Vinokuroff T, Trayhern L (2005). Interpersonal vulnerability to depression in high-risk children: the role of insecure attachment and reassurance seeking. J Clin Child Adolesc Psychol.

[25] Kuruvilla A, Jacob KS (2007). Poverty, social stress & mental health. Indian J Med Res.

[26] Saha S, Hossain MT (2017). Evaluation of medicines dispensing pattern of private pharmacies in Rajshahi. Bangladesh BMC Health Serv Res.

[27] Biswas M, Roy MN, Manik MIN, Hossain MS, Tapu SMTA, Moniruzzaman M, Sultana S (2014). Self medicated antibiotics in Bangladesh: a cross-sectional health survey conducted in the Rajshahi City. BMC Public Health.

[28] Misbahuddin M, Hossain MS, Iqbal KM, Hague MZ (2001). Medical use of opioids in Bangladesh. Lancet.

[29] Hornik R, Yanovitzky I (2003). Using theory to design evaluations of communication campaigns: the case of the National Youth Anti-Drug Media Campaign. Commun Theory.

[30] Wakefield MA, Loken B, Hornik RC (2010). Use of mass media campaigns to change health behaviour. Lancet.

[31] Kwankye SO, Augustt E (2007). Media exposure and reproductive health behaviour among young females in Ghana. Afr Popul Stud.

[32] Intersex campaign for equality. From Wikipedia, the free encyclopedia. https://en.wikipedia.org/wiki/Intersex_Campaign_for_Equality (Last Accessed 21 Sept 2019).

