# Supplementary material for: Barriers in access to healthcare services for individuals with disorders of sex differentiation in Bangladesh: an analysis of regional representative cross-sectional data
Source: BMC Public Health. 2020 Aug 18;20:1261. doi: 10.1186/s12889-020-09284-2 (PMC7437164; doi:10.1186/s12889-020-09284-2)
Supplement: Supplementary file 1 — Additional file 1. DSD questionnaire (DSD population interviewed). Disorder sex development (DSD) population were interviewed using questions of this questionnaire. [file 12889_2020_9284_MOESM1_ESM.pdf]

DSD Questionnaire (DSD population interviewed)

Data Collection Location:

Division:	Region:	Ward:	Ward No:
DSD Community Name:		Location:	

1. Name (DSD):

2. Age (with birth date):

3. Living location:

4. Living condition: (Answer may be one or more than one)

**a.** With parents	**b.** With Life partner	**c.** With other DSD population

5. If separated from the family of the origin, reason of separation: (Answer may be one or more than one)

**a.** Discourage from family	**b.** Attraction by other DSD group	**c.** Psychological factor	**d.** Social factor

6. Treatment during childhood: (Answer may be one or more than one)

**a.** With non-degree trained physician	**b.** With diploma physician	**c.** With MBBS physician	**d.** With specialist in hospital or clinic	**e.** With specialist in foreign country

7. Education: (Answer may be one or more than one)

**a.** Illiterate	**b.** Completed primary education	**c.** Completed secondary education	**d.** Completed higher secondary education	**e.** Completed bachelor or higher education

8. How many times tried for treatment in Private clinics during 2015 and 2016:

2015:	2016:

9. How many times succeed to get treatment in Private clinics during 2015 and 2016:

2015:	2016:

10. How many times tried for treatment in Gov. Hospitals during 2015 and 2016:

2015:	2016:

11. How many times succeed to get treatment Gov. Hospitals during 2015 and 2016:

2015:	2016:

12. Reason of choosing Gov. Hospitals:

**a**. Economic	**b.** Feasible distance	**c.** Available physician	**d.** Other

13. Reason of not getting treatment facilities in Gov. hospital/clinics: (Answer may be more than one)

**a.** Non friendly interaction by hospital staff	**b.** Non friendly interaction by physician	**c.** Public fright as they do not want to stand in line before or after DSD person	**d.** Increased public interest as they congregate around DSD person	**e.** Hospital facilitate treatment to either male or female patient only	f. (other, if any)

Data Collected By:	Date of Collection:
