# Supplementary material for: Barriers in access to healthcare services for individuals with disorders of sex differentiation in Bangladesh: an analysis of regional representative cross-sectional data
Source: BMC Public Health. 2020 Aug 18;20:1261. doi: 10.1186/s12889-020-09284-2 (PMC7437164; doi:10.1186/s12889-020-09284-2)
Supplement: Supplementary file 2 — Additional file 2. DSD questionnaire (Medical staff interviewed). Healthcare services provider’s (physicians) perceptions in providing healthcare services to the DSD population were collected using questions of this questionnaire. [file 12889_2020_9284_MOESM2_ESM.pdf]

DSD Questionnaire (Medical staff interviewed)

Data Collection Location:

Region:	Division:	Country: Bangladesh

1. Name of the hospital:

2. Name of the physician:

3. Type of hospital:

**a.** Government	**b.** Private

4. Location of hospital:

5. Does this hospital have any unit to treat DESD population?

**a.** Yes	**b.** No

6. Does DSD population are using this hospital for medical treatment:

**a.** Yes	**b.** No

7. Which of the following difficulties do you seem faced by hospital's staff of government hospitals in providing treatment to DSD population:
(Answer may be one or more)

**a.** Paper documents supplied by hospital authority have treatment option for male or female patient only.	**b.** Some physician do not prefer to treat DSD patients.	**c.** Population of mainstream society gather around DSD patient which affect hospital management.	**d.** DSD patient enter hospital with excess number of companions (DSD persons) which affect hospital management.	**e.** Hospital staff suspect that DSD population enter hospital to ask for charity instead of illness issues.	**f.** (other, if any)

Data Collected By:	Date of Collection:
